# Sex-Specific Association of Left Ventricular Hypertrophy With Rheumatoid Arthritis

**DOI:** 10.3389/fcvm.2021.676076

**Published:** 2021-06-10

**Authors:** Alessandro Giollo, Giovanni Cioffi, Federica Ognibeni, Riccardo Bixio, Angelo Fassio, Giovanni Adami, Giovanni Orsolini, Andrea Dalbeni, Luca Idolazzi, Davide Gatti, Maurizio Rossini, Ombretta Viapiana

**Affiliations:** ^1^Rheumatology Section, Department of Medicine, University of Verona Hospital Trust, Verona, Italy; ^2^Division of Rheumatology, University of Padova, Padua, Italy; ^3^Division of Cardiac Rehabilitation, San Pancrazio Hospital, Trento, Italy; ^4^Internal Medicine and Hypertension Section, Department of Medicine, University of Verona Hospital Trust, Verona, Italy

**Keywords:** gender medicine, left ventricle hypertrophy, cardiovascular medicine, rheumatoid arthritis, heart disease, heart failure, female sex

## Abstract

**Objectives:** Clinical expression of rheumatoid arthritis (RA) varies by gender, but whether cardiovascular disease (CVD) is gender related in RA is unknown. Left ventricular (LV) hypertrophy (LVH) is a hallmark of CVD in RA patients. We investigated whether the association of LVH with RA is gender driven.

**Methods:** Consecutive outpatients with established RA underwent echocardiography with measurement of LVH at baseline and one follow-up. All participants had no prior history of CVD or diabetes mellitus. We assessed CVD risk factors associated with LVH at follow-up, including sex, age, arterial blood pressure, and body mass index (BMI). We also evaluated inflammatory markers, autoimmunity, disease activity, and the use of RA medications as predictors of LVH.

**Results:** We recruited 145 RA patients (121 females, 83%) and reassessed them after a median (interquartile range) of 36 months (24–50). At baseline, women were more dyslipidemic but otherwise had fewer CVD risk factors than men, including less prevalent smoking habit and hypertension, and smaller waist circumference. At follow-up, we detected LVH in 42/145 (44%) RA patients. LV mass significantly increased only in women. In multiple Cox regression analysis, women with RA had the strongest association with LVH, independently from the presence of CVD risk factors (OR, 6.56; 95% CI, 1.34–30.96) or RA-specific characteristics (OR, 5.14; 95% CI, 1.24–21.34). BMI was also significantly and independently associated with LVH.

**Conclusion:** Among established RA patients, women carry the highest predisposition for LVH.

## Introduction

Rheumatoid arthritis (RA) is a systemic, immune-mediated disease involving both musculoskeletal and extra-articular domains. Cardiovascular disease (CVD) is one of the most common extra-articular manifestations of RA, which can manifest early with abnormalities in left ventricular (LV) geometry and LV hypertrophy (LVH) (1, 2). In particular, concentric LV remodeling is common among RA patients. This association remained significant after adjustment for CVD risk factors and comorbidities (3), suggesting that RA-intrinsic factors could be significantly related to the susceptibility of LVH.

LVH is a risk factor for coronary heart disease (CHD) and poor CVD outcomes in the general population (4), as well as in several settings of patients at increased risk for CVD events (4–8), including RA patients. Several mechanisms, including long-term pressure, such as systemic hypertension or aortic stenosis, can cause LVH. The findings that LVH may precede hypertension and that patients with similar degrees of hypertension may have marked differences in LV mass strongly suggest that genetic and gender-related factors can promote and retard the development of LVH (9). Gender also leads to a predisposition to RA. The incidence of this condition is twice higher in females than males, and disease severity or treatment response differs according to gender (10). However, it is unknown whether susceptibility to LVH in RA patients is gender driven.

In this study, we sought to test the hypothesis that gender is the RA-associated factor most strictly associated with LVH.

## Materials and Methods

### Study Population

The study population included non-institutionalized subjects >18 years of age with RA diagnosed according to the 2010 ACR/EULAR classification criteria. The design of the study was observational prospective. Participants were consecutively recruited from March 2014 to March 2016 at the Division of Rheumatology, Department of Medicine, University and Azienda Ospedaliera Universitaria Integrata of Verona (Italy). They underwent clinical, laboratory, and echocardiographic evaluations at baseline and at follow-up [median (interquartile range), 36 months (24–50)] as part of a CVD primary prevention program. Patients with known CVD including valvulopathies and primary cardiomyopathy have been excluded from this study. To overstate the differences between RA patients with the LVH phenotype and those without, considering the changes in LV mass over time, we divided patients into two groups according to the LVH status at follow-up compared with baseline. Accordingly, we defined “LVH” all participants who had LVH at follow-up, irrespective of LVH status at baseline. Thus, this group comprised patients with persistent and *de novo* LVH. Conversely, the “non-LVH” group included individuals who had no LVH at follow-up, hence, including both patients who had no LVH at baseline and follow-up and those in whom LV mass normalized overtime. All patients gave written informed consent signing a specific institutional consent form. The study was approved by the institutional review board of the University of Verona (1707CESC) and conformed to the ethical guidelines of the Declaration of Helsinki as revised in 2000.

### Cardiovascular Disease Risk Factors

The following CVD risk factors were collected, namely, age; gender; systolic blood pressure, diastolic blood pressure, and heart rate; body mass index (BMI); lipids including total cholesterol, low- and high-density cholesterol, and triglycerides; waist circumference; and renal function. Brachial blood pressure was measured following the European Society of Hypertension guidelines. We used validated oscillometric or auscultatory semiautomatic sphygmomanometers with all patients kept at 5-min rest in a sitting position (11). The average of the last two measurements was taken as the clinic blood pressure. Hypertension was defined as a history of hypertension, use of antihypertensive medication, or elevated blood pressure (systolic blood pressure ≥140 mmHg or diastolic blood pressure ≥90 mmHg) at the clinic visit. We defined obesity when body mass index (BMI) ≥30 kg/m^2^. Dyslipidemia was defined as levels of total serum cholesterol >190 mg/dl and or triglycerides >150 mg/dl or pharmacologically treated high lipid serum levels. To assess renal function, we considered the glomerular filtration rate (GFR) estimated with the CKD-EPI equation.

### Rheumatoid Arthritis-Associated Factors

Data on disease duration, anticitrullinated peptides antibodies (ACPA), and rheumatoid factor (RF) were collected at baseline. Serum biomarkers of RA-related inflammation (C-reactive protein CRP and ESR) were measured. RA disease activity was evaluated by the clinical disease activity index (CDAI) score (12). Current immunomodulating therapies including conventional synthetic disease-modifying antirheumatic drugs (csDMARDs) and biologic DMARDs (bDMARDs), glucocorticoids use and dose and non-steroidal anti-inflammatory drugs (NSAIDs) were recorded.

### Echocardiography

All Doppler-echocardiographic studies were performed using Alpha Esaote Biomedica machine (Florence, Italy) following a standardized protocol by experienced cardiologists. LV chamber dimensions and wall thicknesses were measured by the American Society of Echocardiography guidelines and LV mass was calculated using a validated formula (13). LV mass was normalized for height to the 2.7 power, and LV hypertrophy was defined as LV mass >49.2 g/m^2.7^ for men and >46.7 g/m^2.7^ for women (14). Relative wall thickness was calculated as the ratio 2^*^end-diastolic posterior wall thickness/LV diameter and indicated concentric LV geometry if >0.43 (the 97.5 percentile in a normal population) (15). LV end-diastolic and end-systolic volumes were measured by the biplane method of disks from 2D apical 4 and 2 chamber view and used to calculate LV ejection fraction (LVEF). Assessment of LV diastolic function was based on widely accepted diastolic function parameters, and LV diastolic dysfunction was diagnosed using validated cutoffs of prognostic relevance, as previously reported (16).

### Statistical Analysis

Data are reported as mean values ± standard deviation (medians and interquartile ranges for non-normally distributed variables) or percentages. The study population was stratified by LVH at follow-up. Between-group comparisons of categorical and continuous variables were performed by χ^2^-test and independent samples Student's *t*-test, as appropriate. Longitudinal changes of echocardiography measures were analyzed with the paired-sample *t*-test. Cox regression was run to identify the factors independently related to LVH. Variables that were significantly related to LVH at follow-up in univariable tests (*p* < 0.05) were included in the multivariable logistic regression analysis. In order to avoid overfitting, only the following variables were included in the multivariable model: sex, age, BMI, and SBP. All analyses were performed using the statistical package SPSS 20.0 (SPSS Inc., Chicago, Illinois), and statistical significance was determined by two-tailed *p* < 0.05.

## Results

### Patient Disposition

The study population consisted of 145 white RA patients consecutively enrolled in the study with >1 follow up visit. Treatment included methotrexate in 48%, bDMARDs in 59%, and glucocorticoid therapy in 58% (90% of patients were taking prednisone-equivalent ≤ 5 mg daily); nearly one-third were exposed to NSAIDs occasionally during the 3 months before baseline, but none were chronic NSAID users. Disease activity was moderate or high in 38%. Patients had a median of 2 CVD risk factors, and at least one was present in 92%. A history of current or prior smoking was found in 44%; hypertension was diagnosed in 40%, of whom 92% were on active treatment; dyslipidemia was present in 66% of whom 41% was on treatment with statins; obese patients were 12%; and metabolic syndrome was diagnosed in 11%. No patient had diabetes mellitus.

### Baseline Characteristics of Study Patients According to Gender

LVH was detected in similar proportions (36.4 vs. 33.3%, *p* = 0.777) between women and men at baseline. Concentric LV remodeling was found in a non-significantly higher proportion of women compared with men (28.1 vs. 20.8%; *p* = 0.463). Women had significantly lower LV-EDV and LV-ESV (both *p* < 0.001) and non-significantly lower CI (*p* = 0.096) than men, though all patients had normal LV function and LV volumes. Diastolic dysfunction was found in 26.4% of women and 33.3% of men, respectively (*p* = 0.490). Differences in CVD risk factors and RA charactheristics between males and females are described in Table 1. Women were also more frequently dyslipidemic and on lipid-lowering treatment than men and had significantly shorter waist circumference. Blood pressure levels and medications did not differ between sexes. With regard to RA-specific characteristics, disease activity (CDAI) was higher in women who also used MTX less frequently than men. Inflammatory markers (ESR and CRP) did not differ significantly.

**Table 1:** Baseline characteristics of RA patients according to gender.

**Variables**	**Male RA patients** **(*n* = 24)**	**Female RA patients** **(*n* = 121)**	***P*-value**
**CVD risk factors**			
Age (years)	58.0 (46.0)	59.3 (61.0)	0.628
Body mass index (kg/m^2^)	25.7 (12.3)	24.6 (28.1)	0.493
Waist circumference (cm)	98.0 (39.0)	90.0 (80.0)	0.002
Hypertension (%)	60.9	46.3	0.199
Systolic blood pressure (mmHg)	131.0 (70.0)	130.0 (100.0)	0.873
Diastolic blood pressure (mmHg)	80.0 (40.0)	80.0 (50.0)	0.531
Use of antihypertensives (%)	63.6	44.2	0.096
ACEi/ARBs	54.5	24.8	0.005
Beta-blockers	21.1	13.6	0.565
Calcium-channel blockers	9.1	7.1	0.999
Smoking status (ever, %)	82.6	36.7	<0.001
Dyslipidemia (%)	54.2	67.8	0.241
Use of statins (%)	18.2	28.4	0.319
Metabolic syndrome (%)	16.7	9.9	0.473
eGFR (ml/min/1.73/m^2^)	103.5 (12.3)	91.5 (98.0)	0.112
**RA-specific characteristics**			
C-reactive protein (mg/L)	1.6 (20.9)	1.6 (67.7)	0.539
ESR (mm/h)	7.8 (72.0)	17.8 (75.0)	0.160
Rheumatoid factor (%)	52.4	52.7	0.977
ACPA (%)	61.9	54.6	0.539
Disease duration (years)	14.0 (33.0)	14.0 (49.0)	0.466
CDAI	5.0 (25.0)	9.0 (41.0)	0.078
Current biologic DMARD use (%)	65.2	79.6	0.135
Current MTX use (%)	73.9	42.2	0.006
Current glucocorticoid therapy (%)	63.6	55.6	0.485
Current NSAIDs use (%)	26.1	28.7	0.800

*ACEi, ace inhibitors; ACPA, anticitrullinated peptide antibodies; ARBs, angiotensin II receptor blockers; CDAI, clinical disease activity index; CRP, c-reactive protein; DMARDs, disease-modifying antirheumatic drugs; ESR, erythrocyte sedimentation rate; LV, left ventricular; LVH, left ventricular hypertrophy; NSAIDs, non-steroidal anti-inflammatory drugs; TNF, tumor necrosis factor. Data are reported as median (IQR)*.

### Female Sex Is Associated With LVH in RA

**Figure 1:**
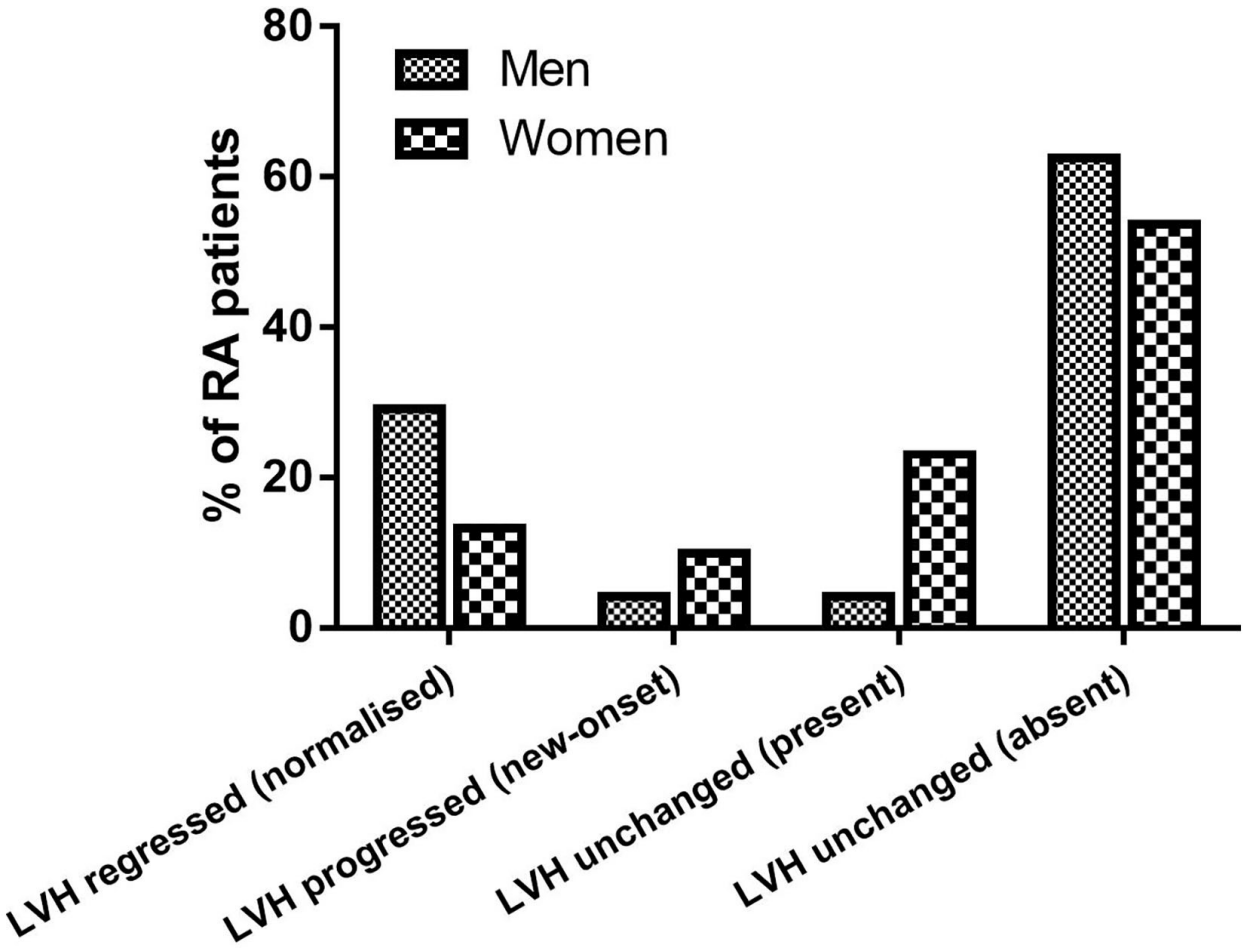
Proportions of RA patients showing LVH regression, LVH progression, and stable LVH at follow-up.

At follow-up, there were 42/145 RA patients with LVH, of whom 13/45 had new-onset LVH. We found a significantly higher proportion of women who had LVH compared with men (40/121 vs. 2/24, 33 vs. 8%, *p* = 0.015), and a nonsignificant higher proportion of new-onset LVH in women than men (12/121 vs. 1/24, 9.9 vs. 4.2%, *p* = 0.695). More women progressed to or remained with LVH while LVH normalized in a higher proportion of men (Figure 1). Women with RA had significantly decreased LV volumes and slightly reduced LV EF and significantly increased LVMI (Table 2). We then tested in cox regression analysis whether female sex was independently associated with LVH. In univariable analysis, the female sex had the strongest association with LVH, followed by age, BMI, waist circumference, blood pressure levels, and renal function. In multivariable analysis, female sex was still independently associated with LVH along with BMI and SBP (Table 3). A second model was run, including only RA-specific factors. CRP was associated with LVH at univariable analysis, but statistical significance was lost after adjusting for gender (Table 4).

**Table 2:** Changes in echocardiography measures at follow-up.

	**Male RA patients (*****n*** **= 24)**					**Female RA patients (*****n*** **= 121)**				
	**Baseline**	**Follow-up**	**MD**	**95% CI**	***P-* value**	**Baseline**	**Follow-up**	**MD**	**95% CI**	***P-* value**
LV EF (%)	64.5 (4.7)	62.7 (5.5)	−1.9	−7.8, 4.1	0.476	67.7 (6.4)	62.3 (4.2)	−5.4	−4.5, −0.2	0.030
LVM/BSA (g/m^2^)	108.9 (19.3)	100.0 (17.9)	−8.9	−0.5, −17.4	0.039	93.4 (20.4)	95.2 (21.6)	1.8	−5.7, 1.9	0.335
LVMi (g/h^2.7^)	43.3 (10.5)	43.0 (11.5)	−0.3	−1.4, 2.0	0.737	43.0 (6.7)	46.9 (9.2)	3.9	0.5, 7.3	0.028
LV septum (cm)	1.14 (0.13)	1.05 (0.10)	−0.09	−0.03, 0.05	0.661	1.03 (0.15)	0.99 (0.16)	−0.04	−0.06, 0.03	0.571
LV EDD/BSA (cm)	2.5 (0.3)	2.5 (0.2)	0.02	−0.06, 0.11	0.604	2.7 (0.3)	2.7 (0.3)	−0.07	−0.1, −0.02	0.009
LV ESD/BSA (cm)	1.6 (0.3)	1.5 (0.3)	−0.10	−0.03, 0.23	0.111	1.6 (0.2)	1.7 (0.3)	−0.04	−0.1, 0.02	0.152
LV EDV (ml)	103.9 (21.0)	104.2 (25.3)	0.3	−10.1, 9.5	0.951	81.9 (20.9)	76.3 (18.2)	−5.6	−9.4, −1.8	0.004
LV ESV (ml)	36.3 (10.1)	39.5 (10.7)	3.3	−8.4, 1.8	0.192	28.8 (14.3)	24.8 (7.7)	−4.0	−6.4, −1.5	0.002
E/A	0.95 (0.28)	0.91 (0.28)	−0.04	−0.05, −0.01	0.331	0.94 (0.32)	0.89 (0.27)	−0.05	−0.0, 0.11	0.071

*BSA, body surface area; E/A, ratio between early and late maximal velocity of left ventricular filling (transmitral flow pattern, pulse wave technique); EDD, end-diastolic diameter; EF, ejection fraction; ESD, end-systolic diameter; LV, left ventricular; LVM, left ventricular mass; LVMi, left ventricular mass index*.

**Table 3:** CVD risk factors significantly associated with the presence of LVH at follow-up: univariable and multivariable Cox regression analyses.

	**Univariable**			**Multivariable**		
	**OR**	**95% CI**	***P***	**OR**	**95% CI**	***P***
Female sex	4.162	1.005–17.231	0.049	6.557	1.389–30.963	0.018
Age (years)	1.062	1.027–10.097	<0.001	1.038	0.996–1.083	0.079
BMI (kg/m^2^)	1.086	1.033–1.141	0.001	1.172	1.063–1.292	0.001
Waist circumference (cm)	1.016	0.992–1.040	0.187			
Systolic blood pressure (mmHg)	1.027	1.013–1.041	<0.001	1.029	1.005–1.054	0.016
Diastolic blood pressure (mmHg)	1.037	1.006–1.068	0.017			
Use of antihypertensives	1.720	0.892–3.316	0.106			
ACEi/ARBs	1.078	0.559–2.078	0.822			
eGFR (ml/min/1.73 m^2^)	0.981	0.964–0.999	0.035			
Dyslipidemia	1.024	0.537–1.954	0.942			
Use of statins	0.991	0.481–2.042	0.981			
Metabolic syndrome	1.541	0.680–3.495	0.300			
Smoking history	0.988	0.530–1.840	0.969			

*ACEi, ACE inhibitors; BMI, body mass index; DBP, diastolic blood pressure; eGFR, estimated glomerular filtration rate*.

**Table 4:** RA-specific variables significantly associated with LVH: univariable and multivariable Cox regression analyses.

	**Univariable**			**Multivariable**		
	**OR**	**95% CI**	***P***	**OR**	**95% CI**	***P***
Female sex	4.385	1.059–18.160	0.042	5.140	1.238–21.337	0.024
CRP (mg/L)	1.025	1.004–1.047	0.022	1.019	0.997–1.041	0.089
ESR (mm/h)	1.007	0.993–1.021	0.339			
Rheumatoid factor	1.237	0.647–2.366	0.519			
ACPA	1.383	0.700–2.731	0.351			
Disease duration (years)	0.997	0.967–1.028	0.832			
CDAI	1.017	0.981–1.053	0.365			
Current MTX use	0.534	0.272–1.049	0.069			
Current glucocorticoid therapy	1.429	0.712–2.866	0.315			
Current biologic DMARD therapy	0.871	0.352–2.152	0.765			

*BMI, body mass index; CRP, C-reactive protein; DBP, diastolic blood pressure; ESR, erythrocyte sedimentation rate; eGFR, estimated glomerular filtration rate; SBP, systolic blood pressure*.

## Discussion

To our knowledge, the present study is the first one to show that LVH associates with gender in RA patients. RA has a female predominance, and several gender-specific factors have been associated with the presence of RA and disease activity. Clinical expression of RA varies by sex, with women less likely than men to develop extra-articular features such as subcutaneous nodules and interstitial lung disease (17, 18). However, it has never been reported that LVH is more represented in women with RA than men.

While it was already established that RA could impair myocardial structure (3), different patterns of heart remodeling across sexes could explain why women with RA progress to LVH more likely than men. We found that women had significantly smaller LV volumes than men at baseline and over time and that LVMI increased during follow-up. We previously showed that patients typically show abnormal concentric LV remodeling compared with matched controls (19). Herein, we observed that this pattern is more characteristic of women. Our findings have clinical relevance since women free of heart disease but with higher LVMI and more LVH at echocardiography are at higher risk of acute myocardial infarction, heart failure, and CV death (20).

This association between LVH, changes in LV geometry, and gender could be related primarily to non-traditional CVD risk factors that are RA disease related (21). Disease duration is independently related to LV mass, suggesting a pathophysiological link between chronic inflammation and LVH (1). An overall greater systemic inflammation due to the disease could justify the larger burden of LVH in women with RA. We observed that women with RA used MTX less frequently than men, and concordantly, higher CRP levels were associated with LVH. Our results agree with other studies reporting that markers of RA chronicity such as disease duration, damage, and CRP are related to LV remodeling (3) and LV mass (1).

Gender is a non-modifiable CVD risk factor. However, BMI was also associated with LVH and independently from gender. Our findings show that bodyweight control is an important outcome in RA patients. In keeping with our observations, excessive body weight was associated with poor disease control and unfavorable CVD outcomes in RA patients (22). In patients with well-controlled, established RA, obesity, and total fat mass are also associated with more inadequate control of inflammation from diagnosis (23).

LVH is usually the response to a chronic pressure or volume load. The two most common conditions associated with LV pressure or volume overload states are systemic hypertension and aortic or mitral valvulopathy. While valvulopathy was an exclusion criterion for this study, half of the study population was hypertensive. Consistent with this, blood pressure levels were significantly associated with LVH, informing that hypertension is a modifiable CVD risk factor for LVH in RA. However, we show that the influence of gender on LVH in RA patients was independent of hypertension for several reasons. First, the proportion of hypertensive women with RA was lower than men with RA as expected (24). Second, the use of antihypertensives was not significantly associated with LVH, especially ACEi and ARBs, which should be protective. Third, blood pressure levels were not different between groups, suggesting that hemodynamic state at baseline did not differ between women and men. Finally, we observed that 14/54 (26%) women with RA had no hypertension but still progressed to LVH. Hence, we argue that the relationship between female sex and LVH does not depend on a different hemodynamic response to pressure load in women compared with men.

Therefore, we could not explain the high burden of LVH in RA women with an excess of CVD risk factors. While in the general population, LVH also often associated with MetS, dyslipidemia, and smoking, we failed to show similar associations in our RA patients. Nevertheless, there were significant differences between women and men RA patients in terms of those features. Women with RA met the criteria for MetS less frequently and were also far less frequent smokers than men. Females were more frequently dyslipidemic than males, but we did not find a significant association between LVH and lipids or statin use.

Our findings suggest that RA-intrinsic factors and bodyweight in excess concur to developing LVH in women with RA. We acknowledge as a limitation that the effect size of gender on LVH should be assessed in an independent cohort since this study was originally conceived as an exploratory study. However, we tried to overcome the limitations of unmeasured confounders by performing multivariable analyses to control for several factors. The study population was referral based, and the extent to which the data can be generalized to other populations remains to be proven. We also acknowledge that having a single time point to reassess all patients would be advisable. One strength of this study is the inclusion of a sample of RA patients with established disease, ensuring that our findings can apply to the vast majority of chronic RA patients we routinely assess in clinics in real life.

In conclusion, RA patients have a 30% of excess in CVD which is related to RA-specific factors that are still substantially unknown (25). Since LVH is a risk factor for acute hemodynamic decompensation, our data support the knowledge that RA patients have a higher likelihood than the general population to suffer from HF (26). It is likely that gender could have a vital role to determine LVH, not due to conventional risk factors. More attention should be paid to LVH in women with RA as abnormal LV remodeling could be more likely to develop and progress than in men and offset the female sex protection in cardiovascular risk. According to our data, RA has a different impact on LVH in men and women.

## Data Availability Statement

The datasets presented in this article are not readily available because data sharing is not applicable to this article. Please contact the corresponding author for data requests. Requests to access the datasets should be directed to Alessandro Giollo, alessandrogiollo@gmail.com.

## Ethics Statement

All patients gave written informed consent signing a specific institutional consent form. The study was approved by the institutional review board of the University of Verona (1707CESC) and conformed to the ethical guidelines of the Declaration of Helsinki as revised in 2000. The patients/participants provided their written informed consent to participate in this study.

## Author Contributions

AG, GC, and OV provided the conception of the study, literature search, data search and interpretation, drafting the article, and critical revision of the article for important intellectual content. GC and FO performed ultrasound studies. RB, AF, GA, GO, AD, LI, DG, MR, and OV provided data search, interpretation of data, drafting the article, and critical revision of the article for important intellectual content. All authors revised the article critically for important intellectual content and gave final approval of the version to be submitted.

## Conflict of Interest

The authors declare that the research was conducted in the absence of any commercial or financial relationships that could be construed as a potential conflict of interest.
